# Design and Validation of Endophthalmitis Infectivity Measurement Algorithm in Post Cataract Acute Endophthalmitis: EMS Report No. 6

**DOI:** 10.1167/tvst.13.8.10

**Published:** 2024-08-07

**Authors:** Taraprasad Das, Jyotiranjan Sahoo, Akash Belenje, Joveeta Joseph, Suchita Pandey, Aditya Kapoor, Rudvij Pandya, Umesh C. Behera, Vivek P. Dave

**Affiliations:** 1 Anant Bajaj Retina Institute-Srimati Kanuri Santhamma Centre for Vitreoretinal Diseases, LV Prasad Eye Institute, Hyderabad, India; 2 Institute of Medical Science and Sum Hospital, SOA University, Bhubaneswar, India; 3 Jhaveri Microbiology Center, L V Prasad Eye Institute, Hyderabad, India; 4 Anant Bajaj Retina Institute, Vitreoretinal service, LV Prasad Eye Institute, Vijayawada, India; 5 Anant Bajaj Retina Institute, Vitreoretinal service, LV Prasad Eye Institute, Vishkhapatnam, India; 6 Anant Bajaj Retina Institute, Vitreoretinal service, LV Prasad Eye Institute, Bhubaneswar, India

**Keywords:** cataract surgery, endophthalmitis, inflammation score, microbiology, statistical modeling

## Abstract

**Purpose:**

We constructed a clinical clue-based algorithm to identify the microbiology-positive post-cataract surgery endophthalmitis.

**Methods:**

The Endophthalmitis Infectivity Measurement Algorithm (EIMA) was constructed using presenting Snellen vision (Letter score [LS]) and Inflammation Score (IS, from the cornea, anterior chamber, iris, and vitreous). Retrospective data (70% for training; 30% for testing) was fitted into CHAID (Chi-squared Automatic Interaction Detection). EIMA was validated with prospective data. EIMA-categorized disease severity was weighed against the symptom duration to detect infecting micro-organisms.

**Results:**

EIMA was constructed from 1444 retrospective data. The average LS was 6.03 ± 12.11, median IS was 14 (8–24), and culture positivity was 38%. The accuracy and area under the curve of CHAID were 66.36% and 0.642, respectively. EIMA was validated with 175 prospectively collected data. Microbiology positivity (culture + sequencing) was 58.9%. EIMA sensitivity, specificity, and accuracy against microbiology-positive endophthalmitis were 73.7 (95% confidence interval [CI], 64.19–81.96), 81.9 (95% CI, 71.1–90.02), 77.1 (95% CI, 70.20–83.14), respectively. The positive and negative likelihood ratios were 4.08 (95% CI, 2.46–6.67) and 0.32 (95% CI, 0.22–0.45), respectively. There was higher microbial growth in two days or less than in three- to six-day symptom duration (69.9% vs. 28.2%; *P* = 0.018) endophthalmitis. Gram-negative infection was higher in two days or less (55.6% vs. 20.2%; *P* = 0.014), and gram-positive infection was higher in three- to six-day endophthalmitis (62.1% vs. 27.7%; *P* = 0.027).

**Conclusions:**

EIMA identified microbiology-positive endophthalmitis three-quarters of the time.

**Translational Relevance:**

EIMA suggested infectivity and the class of microbial infection could help targeted management of endophthalmitis after cataract surgery.

## Introduction

The Endophthalmitis Vitrectomy Study (EVS)–guided current management strategies of acute endophthalmitis after cataract surgery are microbiological workup of undiluted vitreous, intravitreal antibiotics in all patients and vitrectomy in eyes with poor presenting vision.^1^ If required, the treatment is revised after the culture-susceptibility report is available, usually in three to five days. Two important criteria in decision-making are the culture positivity and the presenting vision. The clinical course and features of culture-negative endophthalmitis (other than the toxic anterior segment syndrome) are likely to have different severity of the symptoms and signs than the culture-positive ones. It is challenging to differentiate culture-positive endophthalmitis from culture-negative endophthalmitis from the presenting vision alone. Poor presenting vision could be due to media opacity, such as corneal edema and inflammatory exudates in the pupillary area.^2^

Like infection, inflammation plays an equally significant role in endophthalmitis. The inflammatory cascade activated by specific toxic effects of the pathogen ultimately determines the final anatomical and functional outcome.^3^ It is unclear whether the most severe damage to the visual function is caused by the infectious process or the host's immune response.^4^ Anticipation of infectivity based on the clinical clues at presentation could help the treating ophthalmologist choose an initial treatment close to the final treatment before the microbiology reports are available. We hypothesize that the presenting vision (a surrogate measure for severity of infection) and the presenting inflammation score (a surrogate measure for inflammation) together are likely to differentiate infectious from non-infectious endophthalmitis.

Using large retrospective data, we constructed the Endophthalmitis Infectivity Measurement Algorithm (EIMA) to identify and differentiate infective from non-infective post-cataract surgery acute endophthalmitis. We validated it with a set of prospective data.

## Subjects and Methods

### Subjects

Retrospective data of consecutive patients treated for acute post-cataract endophthalmitis, used to construct the EIMA, were collected from the electronic medical records of a large tertiary eye care institute spread over three adjoining states in India (Andhra, Odisha, and Telangana). Per the institutional practice, all data were entered by optometrists, ophthalmology residents, and retina fellows in their areas of patient examination, and fellowship-trained retina specialists reconfirmed these findings to make the final diagnosis. The retina specialist determined every patient's inflammation score (described later). All patients received a comprehensive eye examination that included a record of the initiating event, time to symptoms, the presenting symptoms, measurement of visual acuity, slit lamp examination of the anterior segment of the eye, indirect ophthalmoscopy, and, when required, B-scan ultrasonography (10 MHz hand-held probe). All patients were treated per the EVS recommendations and the institution's protocol.^1^^,^^5^ All prospective data were collected from the Endophthalmitis Management Study (EMS) participants. These patients were managed per the approved EMS protocol.^6^ In either analysis, the eyes that had received intracameral antibiotic during cataract surgery were not excluded.

### Ethics Committee Approval

All patients were examined and treated after a written and signed informed consent. The Institutional Ethics Committee approved the study (LV Prasad Eye Institute, Hyderabad, India- LEC-BHR-R-07-22-911). The committee waived the additional written informed consent requirement for this analysis because the study only analyzed the precollected data. All methods were performed by the relevant institutional guidelines and followed the tenets of the Declaration of Helsinki for human research.

### EIMA Variables

Two important variables of EIMA were the presenting vision and presenting inflammation score. The vision was measured using an ETDRS chart placed at 4 m, and the number of letters read was documented as the letter score.^7^ The letter score for vision of hand motions and less was counted as 0. The presenting inflammation was quantified on a scale of 0 to 4 from the clinical features of four ocular tissues (cornea, anterior chamber, iris, and vitreous), as described below.

**Inflammation score (IS)** was measured from the at-presentation clinical status of four ocular tissues: cornea (clarity and abscess), anterior chamber (flare/cell and fibrin/hypopyon), iris (blood vessels, exudates over iris), and vitreous (flare, and opacities) per our earlier used and validated data.^8^ The scoring was done from 0 to 4 in each category, with an additional allowance for poor ocular tissue clarity. The anterior chamber cells/flare and vitreous opacities were graded by the SUN (Standardization of Uveitis Nomenclature)^9^ and MUST (Multicentre Uveitis Steroid Treatment)^10^ criteria.

### Microbiology Workup

The basic microbiology workup included microscopy (Gram, Giemsa, and Calcofluor stains) and culture of vitreous (sheep blood agar, aerobic and anaerobic, 37°C incubation; chocolate agar, aerobic, 37°C incubation; Brain-heart infusion broth, aerobic, 37°C incubation; Thioglycolate broth, aerobic, 37°C incubation; and Sabouraud's and Potato dextrose agar, aerobic, 27°C incubation) and antibiotic susceptibility testing. The significance of microbial growth was established by the institutional and EVS standards.^1^^,^^11^ Culture negatives were those without growth in any culture medium. We used Vitek 2 (bio Merieux, Lyon, France) for the identification of bacteria and yeast, the Kirby Bauer disc diffusion assay using CLSI (Clinical and Laboratory Standards Institute) guidelines, and the E-test or Vitek 2 for antimicrobial susceptibility and minimum inhibitory concentration of the drugs. Fungal cultures were identified by macroscopic culture characteristics and microscopic spore morphology on Lactophenol Cotton Blue wet mount staining. Eubacteria and pan-fungal polymerase chain reaction (PCR) was performed in all samples.

### Construction of Algorithm

The retrospective data were entered into a Microsoft Excel (2007) sheet and analyzed using SPSS version 27 (IBM Corporation, Armonk, New York, USA). Categorical variables were expressed in frequency and percentages; the continuous variables were expressed in mean and standard deviation. The normality of the continuous variables was checked using the Shapiro-Wilks test. Univariate analysis was performed using the χ^2^ test and Mann-Whitney U test for association between two categorical variables and comparison of means between two groups, respectively. A *P* value < 0.05 was considered statistically significant. Sensitivity, specificity, positive and negative likelihood ratio, positive and negative predictive value, disease (infectious endophthalmitis prevalence), and accuracy of EIMA vis-à-vis culture-positive, sequencing-positive, and microbiology-positive endophthalmitis were calculated. Sensitivity and specificity were calculated at IS 12.5 (moderate endophthalmitis) and LS 7.5 (Snellen 20/800-20/640).

After evaluating several decision-making models, we used the CHAID (Chi-squared Automatic Interaction Detection) model. It is a classification method for building decision trees using χ^2^ statistics to identify optimal splits.^12^ We divided the datasets randomly into training and testing datasets. The algorithm was designed with 70% (n = 1011) of the dataset, and the results of the algorithms were tested on the remaining 30% (n = 433) of the dataset. The performance of the model was evaluated using accuracy and area under the curve using the receiver operating characteristic curve. We chose the model that had the highest discriminant ability. We used the IS of the ocular tissues significantly associated with culture-positive endophthalmitis and the presenting LS to determine the EIMA predictability. We added two categories of symptom duration (≤2 and 3–6 days) to the EIMA-positive endophthalmitis to identify the infecting micro-organisms.

## Results

### EIMA Construction

The EIMA was constructed with retrospective data of 1444 consecutive patients treated for acute post-cataract endophthalmitis using the already described institutional protocol.^13^ In this group, 38% (n = 548) were culture positive. The mean age was 58.47 ± 16.67 years, the IS was 24.65 ± 20.82, and the mean LS was 6.03 ± 12.11. The sensitivity of IS (@12.5) was higher than LS (@7.5), and the specificity of LS was higher than IS (Table 1).

**Table 1.** Salient Variables in Culture-Positive Acute Post-Cataract Endophthalmitis

Variables	Postoperative (n = 1444)
Culture positive (%)	548 (38.0%)
Average Age	58.47 ± 16.67
Male	286 (54.4%)
Urban habitat	330 (60.2%)
Total Inflammatory Scores	24.65 ± 20.82
Median inflammatory score	14 (8–24)
PVA	
Snellen LP- ≤ 20/400. n (%)	507 (92.9)
Average letter scores	6.03 ± 12.11
Median Letter score	5 (0–10)
Sensitivity (%)	
Inflammation score % (mean, 54.06)	@12.5:67.3
Letter score % (mean, 35.96)	@ 7.5:36.9
Specificity (%)	
Inflammatory score (%) (mean, 59.5)	@12.5:52.3
Letter score (%) (mean, 79.5)	@ 7.5:75.9

We analyzed the total and individual IS vis-à-vis the culture results. In univariate analysis, cornea clarity, corneal abscess, anterior chamber hypopyon, exudates over the iris, and vitreous opacity scores were significantly higher in culture-positive endophthalmitis. The mean total IS was significantly higher in culture-positive patients (24.65 ± 20.82) than in culture-negative patients (17.86 ± 18.14; *P* < 0.001). The IS was statistically higher in all individual tissue lesions except for the anterior chamber cells and iris vessel dilation. The six factors significantly associated with culture-positive endophthalmitis were cornea clarity and abscess, anterior chamber hypopyon, exudates over the iris, vitreous opacities and flare, and higher inflammation score (Table 2).

**Table 2.** Comparison of Mean of Individual Inflammation Scores in Culture-Positive and -Negative Postoperative Endophthalmitis

	Culture-Negative		Culture-Positive (n = 548; 38%)		Total (n = 1444)		
Variables	Mean ± SD	Median (IQR)	Mean ± SD	Median (IQR)	Mean ± SD	Median (IQR)	*P*
Cornea clarity score	3.47 ± 6.32	2 (1–3)	5.53 ± 7.98	3 (2–3)	4.25 ± 7.07	2 (1–3)	<0.001*
Cornea abscess score	0.73 ± 2.61	0 (0–0)	1.32 ± 3.02	0 (0–2)	0.95 ± 2.79	0 (0–0)	<0.001*
AC cells score	2.13 ± 1.58	2 (0–4)	2.08 ± 1.65	2 (0–4)	2.11 ± 1.61	2 (0–4)	0.776
AC hypopyon score	3.65 ± 5.93	0 (0–2)	3.63 ± 5.87	2 (0–3)	2.67 ± 5.20	1 (0–2)	<0.001*
Iris exudates score	3.63 ± 5.88	0 (0–3)	5.16 ± 6.36	1 (0–14)	4.21 ± 6.11	0 (0–14)	<0.001*
Iris vessel dilation score	0.61 ± 2.78	0 (0–0)	0.90 ± 3.39	0 (0–0)	0.72 ± 3.02	0 (0–0)	0.108
Vitreous flare score	1.54 ± 1.66	1 (0–3)	1.36 ± 1.66	0 (0–3)	1.47 ± 1.67	0 (0–3)	0.027*
Vitreous opacity score	3.70 ± 3.74	2 (0–9)	4.68 ± 3.99	4 (0–9)	4.07 ± 3.87	2 (0–9)	<0.001*
Inflammation score (Total)	17.86 ± 18.14	12 (7–21)	24.65 ± 20.82	17 (11–31)	20.44 ± 19.47	14 (8–24)	<0.001*

AC, anterior chamber; IQR, interquartile range; PVA, presenting visual acuity; SD, standard deviation; OR, odds ratio.

* Significant (Mann-Whitney test).

The accuracy of the CHAID model used to construct the EIMA was 64.32%, and the area under the curve was 0.642. (Supplementary Fig. S1) Supplementary Table S1 shows various parameters in the EIMA testing data set (n = 433) in culture-positive endophthalmitis. Table 3 shows the sensitivity, specificity, positive and negative likelihood ratio, and positive and negative predictive value of EIMA against culture-positive, PCR-positive, and microbiology-positive endophthalmitis. We validated EIMA with prospectively collected microbiology data of 175 people treated for acute post-cataract endophthalmitis between April 2019 and September 2022. Culture-positive (all of them were also PCR-positive) was 36.0% (63 of 175), and culture-negative + PCR-positive was 22.9% (40 of 175). Thus microbiology positivity was 58.9% (103 of 175).

**Table 3.** EIMA in Post-Cataract Endophthalmitis

	Culture +ve & PCR +ve (n = 63/175; 36.0%)		PCR +ve; & Culture −ve (n = 40/175; 22.9%)		Micro +ve (n = 103/175; 58.9%)	
Parameters	Value	95% CI	Value	95% CI	Value	95% CI
Sensitivity	82.5	70.0–90.9	60.0	43.3–75.1	73.7	64.19–81.96
Specificity	81.9	71.1–90.0	81.9	71.1–90.0	81.9	71.1–90.02
Positive likelihood ratio	4.5	2.7–7.5	3.23	1.91–5.77	4.08	2.46–6.67
Negative likelihood ratio	0.2	0.12–0.36	0.48	0.32–0.72	0.32	0.22–0.45
Disease prevalence	46.6	38.03–55.44	35.7	26.8–45.3	58.8	51.18–66.2
Positive predictive value	80.0	70.70–86.89	64.8	51.49–76.25	85.3	77.91–90.64
Negative predictive value	84.2	75.61–90.26	78.6	71.30–84.54	68.6	60.82–75.46
Accuracy	82.2	74.71–88.26	74.1	64.97–81.23	77.1	70.20–83.14

PCR, polymerase chain reaction.

#### Clinical Algorithm

Using the presenting IS and LS, there were six possible situations, three each for microbiology-positive and microbiology-negative endophthalmitis (Fig.).

**Figure.**
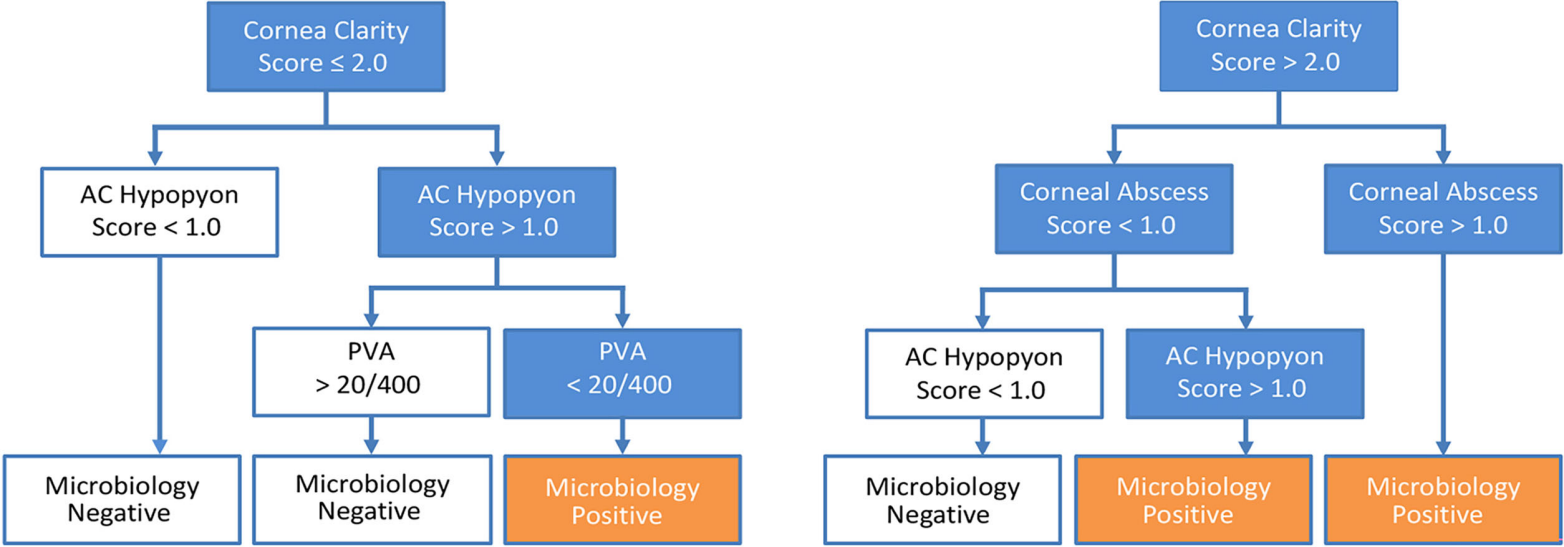
Clinical algorithm to identify post-cataract microbiology-positive endophthalmitis.

Three situations for microbiology-positive end-ophthalmitis:

1. Corneal edema IS ≤ 2 (iris visible) + hypopyon IS > 1 (>25% of anterior chamber) + presenting vision < 20/400
2. Corneal edema IS > 2 (iris barely visible) + corneal abscess IS ≤ 1 (measuring < 1 mm) + hypopyon IS > 1 (>25% of anterior chamber)
3. Corneal edema IS > 2 (iris barely visible) + Corneal abscess IS > 1 (measuring 1–2 mm)

Three situations for microbiology-negative end-ophthalmitis:

1. Corneal edema IS ≤ 2 (iris visible) + hypopyon IS ≤ 1 (trace)
2. Corneal edema IS ≤ 2 (iris visible) + hypopyon IS > 1 (<25% of anterior chamber) + presenting vision > 20/400
3. Corneal edema IS > 2 (iris barely visible) + Corneal abscess ≤ 1 (measuring < 1 m) + hypopyon IS ≤ 1 (trace)

Two situations in either case above were independent of presenting vision.

In building the clinical algorithm, only the anterior segment findings seen in the slit lamp were included because these findings were imaged in all the eyes; the vitreous conditions (opacities and flare) were excluded because these were not imaged in all the eyes.

### EIMA Validation

The EIMA was validated with prospectively collected data from 175 consecutive post-cataract endophthalmitis. The microbiology-positive group consisted of 35 (34%) gram-positive cocci, 48 (46.6%) gram-negative bacilli, 19 (18.4%) fungi, and one (1%) gram-positive bacillus. There was a difference between the culture and Sanger sequencing results. The proportion of culture and Sanger-positive micro-organisms were 33.3% and 35%, respectively, for gram-positive cocci, 55.6% and 32.8% for gram-negative bacilli, and 9.5% and 32.5% for fungi.

The sensitivity, specificity, positive- and negative likelihood ratio, and positive and negative predictive value of EIMA against culture-positive, PCR-positive, and microbiology-positive endophthalmitis are shown in Table 3. The accuracy of EIMA was 82.2% (95% CI, 74.71–88.26), 74.1 (95% CI, 64.97–81.23), and 77.1 (95% CI, 70.20–83.14), respectively (Table 3).

*Culture-positive (also PCR positive)*: The EIMA accuracy was 82.5% (52 of 63). EIMA failed to predict in 11 instances. The presenting vision was >20/400 in two of 11 eyes; the cornea was clear in three of 11 eyes; none of these eyes had a corneal abscess, trace hypopyon (grade <1) was present in nine of 11 eyes, and the IS was ≥10 (11–16) in nine of 11 eyes. Vitreous grew gram-positive cocci (*Staphylococcus* species), gram-negative bacilli (chiefly, *Pseudomonas* species), and gram-positive bacilli (*Kocuria* species) in six (54.5%), four (36.4%), and one eye, respectively (Table 4).

*Culture-negative + PCR positive*: The EIMA accuracy was 60.0% (24 of 40). EIMA failed to predict in 16 instances. The presenting vision was >20/400 in four of 16 eyes; the cornea was clear in 12 of 16 eyes; none of these eyes had a corneal abscess, 14 of 16 eyes had trace hypopyon (grade <1), and IS was ≥10 (10–16) in 11 of 16 eyes (Table 4).

*Culture-negative + PCR negative*: The EIMA false positivity was 18% (13 of 72). EIMA failed to predict in 13 instances. The presenting vision was <20/400 in 100 % (13 of 13) of eyes, 92.3% (12 of 13) eyes had corneal edema, 69.2% (nine of 13) of eyes had a corneal abscess, and 100% (13 of 13) eyes had hypopyon (Table 4).

Thus EIMA predicted microbiology-positive results 73.8% (76 of 103) of the time, the false-positive was 18%, and it was undecided 8.2% of the time. It invariably failed when the presenting vision was >20/400 or there was trace or no hypopyon.

### EIMA Algorithm Versus Symptom Duration and Microorganisms

We measured the micro-positive endophthalmitis against the duration of symptoms. There was higher microbial growth in two days or less than in three- to six-day symptom duration (69.9% vs. 28.2%; *P* = 0.018). The eyes that developed symptoms early had more gram-negative bacterial infection (≤2 days: 69.9% of micro-positive endophthalmitis; 55.6% gram-negative and 20.8% gram-positive bacteria, *P* = 0.014), and the eyes that developed symptoms in three to six days had more of gram-positive bacterial infection (28.2% of micro-positive endophthalmitis; 62.1% gram-positive cocci, and 27.6% gram-negative bacilli, *P* = 0.027). Also, fungal infection was seen in eyes with shorter than longer symptom duration (22.2% vs. 10.3%, *P* = 0.030). Irrespective of the time to develop the symptom, *Staphylococcus*, *Pseudomonas*, and *Aspergillus* species were the predominating gram-positive, gram-negative, and fungal-infecting micro-organisms, respectively. (Supplementary Table S2)

## Discussion

Endophthalmitis treatment aims to sterilize the eye, arrest inflammation, minimize tissue damage, and rescue functional vision. Although the recommended management includes intravitreal and topical antimicrobials and vitrectomy, an adequate treatment strategy should also consider the nature of the pathogen, such as the causative micro-organisms (bacteria and fungi), potential virulence, and antibiotic susceptibility irrespective of cause (intraocular surgery, trauma and endogenous) and time of presentation (acute, and late-onset). One does not know the infecting organism at presentation, and the empiric treatment in acute post-cataract endophthalmitis is primarily based on the clinical signs and accumulated experience/protocol of the treating physician or the treating center^5^ or the EVS recommendations.^1^

Intraocular inflammation is a critical component of endophthalmitis. Once in the vitreous cavity, the micro-organisms incite inflammation. The biological events and the course depend on the organismal virulence and their toxins. The polymorphonuclear neutrophils, the primary infiltrating cell type in infectious endophthalmitis, liberate toxic oxygen metabolites and proteolytic enzymes that could damage the sensitive retinal tissues (Muller and RPE cells).^3^

In this cohort, positive culture between 36% and 38% (prospective and retrospective data, respectively) was low. Possibly it was because, as a referral center, most of these patients had received intracameral antibiotic during cataract surgery. The microbiology positivity was 58.9% (Table 4) using the newer molecular methods (PCR, Sangers, NGS).^14^^,^^15^ The presenting visual acuity is currently used as the surrogate measure for disease severity, but it may not always accurately indicate the severity of endophthalmitis.^16^ Quantifying inflammation associated with infectious endophthalmitis is equally necessary for deciding on the primary (empiric) treatment. Obtaining it before the culture results are available would be valuable to the treating physician.

**Table 4.** EIMA Versus Microbiology: Description of Cases Where EIMA Did Not Predict Microbiology Positivity

	Culture Positive/PCR Positive (n = 63) PCR- Eubacteria- All 63 EIMA Predictability: 82.5% (52/63)						Culture Negative/PCR Positive (n = 40) PCR: 13 Panfungal, 27 Eubacterial EIMA Predictability: 64.2% (43/67)						Culture Negative/PCR Negative (n = 72) EIMA False Predictability 18% (13/72)				
No.	EIMA negative: n = 11 (7.5%) Culture	VA	CC	CA	Hyp	Total IS	EIMA negative: n = 16 (35.8%) PCR	VA	CC	CA	Hyp	Total IS	VA	CC	CA	Hyp	Total IS
1	*S. epidermidis*	20/250	2	0	0	7	Panfungi	CF	0	0	1	10	LP	4	3	3	20
2	*R. mannitolilytica*	20/200	2	0	1	10	Eubacteria	HM	0	0	0	11	LP	0	0	2	15
3	*S. aureus*	CF	2	0	1	11	Eubacteria	HM	1	0	1	18	LP	2	0	0	16
4	*S. epidermidis*	CF	2	0	1	14	Panfungi	20/60	0	0	1	10	LP	2	0	2	20
*5*	*P. aeruginosa*	20/40	0	0	1	8	Panfungi	HM	1	0	1	11	LP	2	0	2	16
*6*	*S. pseudointermedius*	20/400	1	0	1	12	Panfungi	HM	0	0	1	9	LP	3	1	2	31
*7*	*S. epidermidis*	HM	2	0	1	16	Eubacteria	CF	0	0	1	7	LP	3	1	2	18
*8*	*P. aeruginosa*	HM	2	0	1	16	Panfungi	CF	0	0	1	8	LP	2	0	2	16
9	*P. mendocina*	HM	0	0	1	11	Eubacteria	CF	0	0	1	11	HM	3	0	2	24
10	*S. epidermidis*	HM	0	0	0	16	Eubacteria	HM	0	0	1	11	LP	3	0	2	18
11	*K. kristinae*	20/800	1	0	0	14	Eubacteria	20/160	0	0	1	12	LP	3	1	2	20
12	—						Eubacteria	20/160	0	0	1	16	LP	2	0	2	20
13	—						Eubacteria	20/80	0	0	0	8	LP	3	2	3	20
14	—						Eubacteria	20/800	0	0	0	4	—				
15	—						Eubacteria	HM	1	0	1	11	—				
16	—						Eubacteria	HM	1	0	1	11	—				

CA, corneal abscess; CC, cornea clarity; CF, Count Finger close to face; EIMA, endophthalmitis infectivity measurement algorithm; Hyp, hypopyon; LP, light perception; VA, visual acuity.

We used the cutoff values of the total IS at 12.5 (moderate to severe infection) and the LS at 7.5 (Snellen vision 20/640- 20/800) to calculate the sensitivity and specificity for culture-positive endophthalmitis. The EIMA considered presenting IS and LS (Fig.). The combination of the inflammation score and presenting vision was an important deciding factor when the external signs were less severe. Although the severity of inflammation was always important, the presenting vision was important in milder cases (Fig.).

The EVS suggested vitrectomy in eyes with presenting vision of less than HM (letter score 0).^1^ The EVS also showed that people with better vision benefit from a less invasive and less expensive procedure, such as intravitreal antibiotics alone, even in culture-positive endophthalmitis, and may not always require a vitrectomy. The technical safety of the vitrectomy procedure in the 1990s could be one of the reasons for the decision not to recommend vitrectomy in every eye. Over the past three decades, there has been significant progress in vitreous surgery technology; it is safer, more reliable, has reduced operating time, and often precludes hospitalization. There is also increasing evidence of superior functional outcomes after primary vitrectomy and intravitreal antibiotics in endophthalmitis than intravitreal antibiotics alone.^17^^,^^18^

A decision-making algorithm is a mathematical representation of observed data that builds a relationship between the variables. Currently, no model objectively differentiates between culture-positive and -negative endophthalmitis based on the clinical features. The EVS used presenting vision to measure the disease severity. The current study shows that the Inflammation Score is a good surrogate measure of disease severity in endophthalmitis. It is objective and validated.^19^ An algorithmic assessment using the inflammation score of the ocular tissues showed that the degree of corneal involvement (edema and abscess) and hypopyon height are the major indicators of predicting the microbiology-positive endophthalmitis. Combining with the presenting vision is equally important. It showed that the presenting vision >20/400, even in the presence of hypopyon (<25% of anterior chamber height), would be microbiology-negative endophthalmitis (Fig.). The analysis also showed that eyes developing symptoms within two days of surgery are infected by more virulent micro-organisms (Supplementary Table S2).

### Weakness and Strength

#### Weakness

We analyzed the inflammation score against only culture-positive endophthalmitis, not microbiology-positive, including other laboratory investigations such as PCR, primarily because it was not done in all cases in this cohort of people. Also, at the AUROC discrimination at 64.2%, the ability to differentiate culture-positive from culture-negative endophthalmitis is not high but “acceptable”^20^; in the absence of any such model in endophthalmitis, it is better than pure chance (50%). Our data did not model for fungal endophthalmitis; this might be important in the regions with sizable fungal infection.

The incidence of post-cataract surgery endophthalmitis has considerably reduced, so the sample size (n = 1444) is likely adequate given that these patients were treated using a uniform clinical and microbiology workup protocol. EIMA validation was not done in large eye care facilities in India, although the validation with prospectively collected data from a randomized clinical trial has some merit.

#### Strengths

It is the first study to apply inflammation scores to a large data set of clinical endophthalmitis. It is also the first attempt to design an algorithm to predict the infectivity of endophthalmitis on clinical examinations to guide therapy before the microbiology reports are available. It was highly accurate in culture-positive and PCR-positive endophthalmitis. Combining the presenting inflammation score and vision, the predictability of EIMA for microbiology-positive endophthalmitis (culture + PCR) was 73.8%.

## Clinical Implication and Conclusion

It is agreed that early and appropriate treatment helps restore better vision in endophthalmitis.^21^ However, it also depends on infectivity, infecting micro-organisms, and antibiotic susceptibility.^22^ Treating all patients according to the EVS recommendations is increasingly questioned.^23^ Studies have shown better anatomic and visual outcomes after early and near-complete vitrectomy.^24^^,^^25^ However, vitrectomy is needed only for eyes with infectious endophthalmitis. The proposed algorithm with a higher positive likelihood ratio (4.08) and higher predictive value (77.91%) has a greater ability to detect infectious endophthalmitis (Table 3) in the subjects studied in India. Given its 18% false-positive rate, validating it in different regions of India and the world would be good. Our study shows that using an algorithm that combines inflammation score and presenting vision could identify infectious endophthalmitis three-quarters of the time. The results are for microbiologic positivity and, hence, equally valuable for culture-negative cases. Arguably, the current accuracy could be better, although it could be a good starting point for treating surgeons.

## Supplementary Material

Supplement 1

Supplement 2

Supplement 3
