# Supplementary figures and images for: Design and Validation of Endophthalmitis Infectivity Measurement Algorithm in Post Cataract Acute Endophthalmitis: EMS Report No. 6
Source: Transl Vis Sci Technol. 2024 Aug 7;13(8):10. doi: 10.1167/tvst.13.8.10 (PMC11316448; doi:10.1167/tvst.13.8.10)

**Supplementary Figure 1.** Performance evaluation of CHAID using ROC curve

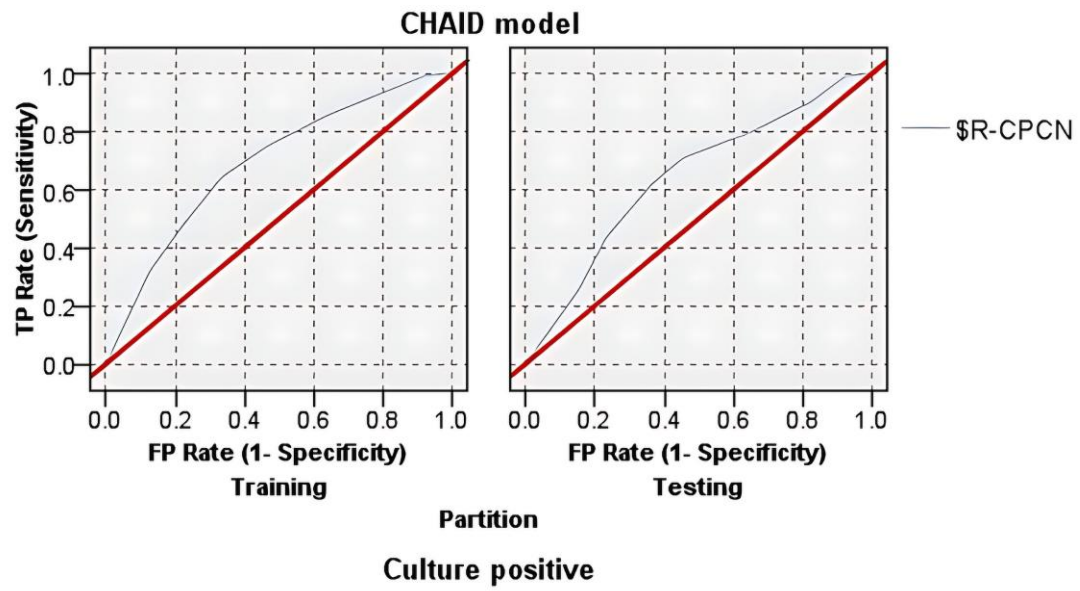

Supplement: Supplement 1 [file tvst-13-8-10_s001.pdf]
