# Supplementary material for: Design and Validation of Endophthalmitis Infectivity Measurement Algorithm in Post Cataract Acute Endophthalmitis: EMS Report No. 6
Source: Transl Vis Sci Technol. 2024 Aug 7;13(8):10. doi: 10.1167/tvst.13.8.10 (PMC11316448; doi:10.1167/tvst.13.8.10)
Supplement: Supplement 2 [file tvst-13-8-10_s002.docx]

**Design and Validation of Endophthalmitis Infectivity Measurement Algorithm in Post Cataract Acute Endophthalmitis. EMS Report # 6**

**Supplementary Table1. Measuring performance of EIMA-in Testing data set (n=433)**

| Parameters | EIMA performance vs Culture +ve | |
| --- | --- | --- |
|  | Value | 95% CI |
| Sensitivity % | 59.7 | 52.3 – 67.1 |
| Specificity % | 65.3 | 59.5 – 71.1 |
| Positive Likelihood Ratio | 1.72 | 1.2 – 3.6 |
| Negative likelihood ratio | 0.62 | 0.34 – 0.78 |
| Disease Prevalence % | 39.5 | 34.9 – 44.2 |
| Positive Predictive Value % | 52.9 | 45.9 – 59.9 |
| Negative Predictive Value % | 71.3 | 65.6 – 77.0 |
| Accuracy | 63.0 | 58.5 – 67.6 |
