# Supplementary material for: Design and Validation of Endophthalmitis Infectivity Measurement Algorithm in Post Cataract Acute Endophthalmitis: EMS Report No. 6
Source: Transl Vis Sci Technol. 2024 Aug 7;13(8):10. doi: 10.1167/tvst.13.8.10 (PMC11316448; doi:10.1167/tvst.13.8.10)
Supplement: Supplement 3 [file tvst-13-8-10_s003.docx]

**Endophthalmitis Infectivity Measurement Algorithm in Post Cataract Acute Endophthalmitis. EMS Report # 6**

**Running title:** **Endophthalmitis Infectivity Measurement Algorithm**

**Supplementary Table 2.** EIMA algorithm measured against symptoms duration and microorganisms (n=103; culture positive= 40 + Sequencing positive = 40))

| Distribution by symptoms days | Organism class | Cornea clarity <2 AC hypopyon >1 PVA <20/400. n= 31 | Cornea clarity > 2  Corneal abscess < 1  AC hypopyon >1.  n= 48 | Cornea clarity >2  Corneal abscess >1.  n= 24 |
| --- | --- | --- | --- | --- |
| < 2 days  n=72 (69.9%)  GPC-15 (20.8%)  **GNB- 40 (55.6%)**  GPB-2 (1.4%)  Fungi- 16 (22.2%)  P GPC (20.8%) vs GNB (55.6%)= 0.014 | Total | n=18 | n= 34 | n= 20 |
|  | GPC  *Staphylococcus aureus-* 5  *Staphylococcus epidermidis-* 5  *Streptococcus oralis-* 3  *Enterococcus species-* 1  *Streptococcus pneumoniae-* 1 | 7 | 8 | 0 |
|  | GNB  *Pseudomonas aeruginosa*- 15  *Pseudomonas stutzeri*- 8  *Sphingomonas paucimobilis*- 3  *Ralstonia mannitolilytica*- 3  *Serratia marcescens*- 3  *Pseudomonas putida*- 2  *Pseudomonas fluorescens*- 2  *Klebsiella species*- 2  *Morgnella morganii*- 1 | 6 | 18 | 16 |
|  | GPB  *Clostridium species*- 1  *Bacillus cereus* 1 | 0 | 1 | 1 |
|  | Fungi  *Fusarium solani*- 7  *Aspergillus flavus*- 5  *Aspergillus nidulans*- 2  *Aspergillus fumigatus*- 1  *Acremonium*-1 | 5 | 7 | 4 |
| 3-6 days  n= 29 (28.2%)  **GPC- 18 (62.1%)**  GNB- 8 (27.6%)  Fungi- 3 (10.3%)  P GPC (62.1%) vs GNB (27.6%)= 0.027 | Total | n= 11 | n= 14 | n= 4 |
|  | GPC  *Staphylococcus aureu*s- 7  *Staphylococcus epidermidis*- 6  *Staphylococcus mitis*- 2  *Streptococcus oralis*- 2  *Kocuria kristinae*- 1 | 5 | 9 | 4 |
|  | GNB  *Pseudomonas aeruginosa*- 3  *Ralstonia mannitolilytica*- 2  *Pseudomonas monteilli*- 1  *Pseudomonas stutzeri*- 1  *Muribaculum species-* 1 | 3 | 5 | 0 |
|  | GPB | 0 | 0 | 0 |
|  | Fungi  *Pencillium rubens*- 1  *Aspergillus flavus*- 1  *Aspergillus Tereus*- 1 | 3 | 0 | 0 |
| >7 days  n=2 (1.9%) | Total | n= 2 | n=0 | n=0 |
|  | GPC  *Staphylococcus epidermidis*- 2 | 2 |  |  |
